# Emerging opportunities of using large language models for translation between drug molecules and indications

**DOI:** 10.1038/s41598-024-61124-0

**Published:** 2024-05-10

**Authors:** David Oniani, Jordan Hilsman, Chengxi Zang, Junmei Wang, Lianjin Cai, Jan Zawala, Yanshan Wang

**Affiliations:** 1 Department of Health Information Management, University of Pittsburgh, Pittsburgh, PA USA; 2 Department of Population Health Sciences, Weill Cornell Medicine, New York, NY USA; 3 Institute of Artificial Intelligence for Digital Health, Weill Cornell Medicine, New York, NY USA; 4 Department of Pharmaceutical Sciences, University of Pittsburgh, Pittsburgh, PA USA; 5 Jerzy Haber Institute of Catalysis and Surface Chemistry, Polish Academy of Sciences, Kraków, Poland; 6 Department of Biomedical Informatics, University of Pittsburgh, Pittsburgh, PA USA; 7 Intelligent Systems Program, University of Pittsburgh, Pittsburgh, PA USA; 8 Clinical and Translational Science Institute, University of Pittsburgh, Pittsburgh, PA USA

**Keywords:** Computer science, Artificial intelligence, Large language models, Drug discovery, Drug discovery, Computational science, Computer science, Scientific data

## Abstract

A drug molecule is a substance that changes an organism’s mental or physical state. Every approved drug has an indication, which refers to the therapeutic use of that drug for treating a particular medical condition. While the Large Language Model (LLM), a generative Artificial Intelligence (AI) technique, has recently demonstrated effectiveness in translating between molecules and their textual descriptions, there remains a gap in research regarding their application in facilitating the translation between drug molecules and indications (which describes the disease, condition or symptoms for which the drug is used), or vice versa. Addressing this challenge could greatly benefit the drug discovery process. The capability of generating a drug from a given indication would allow for the discovery of drugs targeting specific diseases or targets and ultimately provide patients with better treatments. In this paper, we first propose a new task, the translation between drug molecules and corresponding indications, and then test existing LLMs on this new task. Specifically, we consider nine variations of the T5 LLM and evaluate them on two public datasets obtained from ChEMBL and DrugBank. Our experiments show the early results of using LLMs for this task and provide a perspective on the state-of-the-art. We also emphasize the current limitations and discuss future work that has the potential to improve the performance on this task. The creation of molecules from indications, or vice versa, will allow for more efficient targeting of diseases and significantly reduce the cost of drug discovery, with the potential to revolutionize the field of drug discovery in the era of generative AI.

## Introduction

Drug discovery is a costly process^1^ that identifies chemical entities with the potential to become therapeutic agents^2^. Due to its clear benefits and significance to health, drug discovery has become an active area of research, with researchers attempting to automate and streamline drug discovery^3,4^. Approved drugs have indications, which refer to the use of that drug for treating a particular disease, condition, or symptoms^5^. They specify whether the drug is intended for treatment, prevention, mitigation, cure, relief, or diagnosis of that particular ailment. The creation of molecules from indications, or vice versa, will allow for more efficient targeting of diseases and significantly reduce the cost of drug discovery, with the potential to revolutionize the field.

Large Language Models (LLMs) have become one of the major directions of generative Artificial Intelligence (AI) research, with highly performant models like GPT-3^6^, GPT-4^7^, LLaMA^8^, and Mixtral^9^ developed in the recent years and services like ChatGPT reaching over 100 million users^10,11^. LLMs utilize deep learning methods to perform various Natural Language Processing (NLP) tasks, such as text generation^12,13^ and neural machine translation^14,15^. The capabilities of LLMs are due in part to their training on large-scale textual data, making the models familiar with a wide array of topics. LLMs have also demonstrated promising performance in a variety of tasks across different scientific fields^16–19^. Since LLMs work with textual data, the first step is usually finding a way to express a problem in terms of text or language.

An image or a diagram is a typical way to present a molecule, but methods for obtaining textual representations of molecules do exist. One such method is the Simplified Molecular-Input Line-Entry System (SMILES)^20^, which is usually considered as a language for describing molecules. As SMILES strings represent drugs in textual form, we can assess the viability of LLMs in translation between drug molecules and their indications. In this paper, we consider two tasks: *drug-to-indication* and *indication-to-drug*, where we seek to generate indications from the SMILES strings of drugs, and SMILES strings from possible indications, respectively. Translation between drugs and the corresponding indication will allow for finding a cure for diseases that have no current treatment.

Research efforts have attempted de-novo drug discovery through the use of AI, including graph neural networks^21,22^ and, more recently, forms of generative AI^23^. There are numerous existing efforts for molecular design and drug discovery using AI, such as GPT-based models using scaffold SMILES strings accompanied with desired properties of the output molecule^24^. Others have used T5 architecture for various tasks, such as reaction prediction^25^ and converting between molecular captions and SMILES strings^26^. Additional work in the field is centered around the generation of new molecules from gene expression signatures using generative adversarial networks^27^, training recurrent neural networks on known compounds and their SMILES strings, then fine-tuning for specific agonists of certain receptors^28^, or using graph neural networks to predict drugs and their corresponding indications from SMILES^29^. As such, there is an established promise in using AI for drug discovery and molecular design. Efforts to make data more friendly for AI generation of drugs also include the development of the Self-Referencing Embedded Strings (SELFIES)^30^, which can represent every valid molecule. The reasoning is that such a format will allow generative AI to construct valid molecules while maintaining crucial structural information in the string. The collection of these efforts sets the stage for our attempt at generating drug indications from molecules.

With advancements in medicinal chemistry leading to an increasing number of drugs designed for complex processes, it becomes crucial to comprehend the distinctive characteristics and subtle nuances of each drug. In this direction, researchers have released many resources, including datasets that bridge medicines and chemical ingredients like TCMBank^31,32^, models for generating high-quality molecular representations to facilitate Computer-Aided Drug Design (CADD)^33^, and models for drug-drug interactions^34,35^. This has also led to the development of molecular fingerprints, such as the Morgan fingerprint^36^ and the MAP4 fingerprint^37^, which use unique algorithms to vectorize the characteristics of a molecule. Computation of fingerprint representations is rapid, and they maintain much of the features of a molecule^38^. Molecular fingerprinting methods commonly receive input in the form of SMILES strings, which serve as a linear notation for representing molecules in their structural forms, taking into account the different atoms present, the bonds between atoms, as well as other key characteristics, such as branches, cyclic structures, and aromaticity^20^. Since SMILES is a universal method of communicating the structure of different molecules, it is appropriate to use SMILES strings for generating fingerprints. Mol2vec^39^ feeds Morgan fingerprints to the Word2vec^40^ algorithm by converting molecules into their textual representations. Bidirectional Encoder Representations from Transformers (BERT)^41^-based models have also been used for obtaining molecular representations, including models like MolBERT^42^ and ChemBERTa^43^, which are pretrained BERT instances that take SMILES strings as input and perform downstream tasks on molecular representation and molecular property prediction, respectively. Other efforts in using AI for molecular representations include generating novel molecular graphs through the use of reinforcement learning, decomposition, and reassembly^44^ and the prediction of 3D representations of small molecules based on their 2D graphical counterparts^45^.

In this paper, we evaluate the capabilities of MolT5, a T5-based model, in translating between drugs and their indications through the two tasks, drug-to-indication and indication-to-drug, using the drug data from DrugBank and ChEMBL. The drug-to-indication task utilizes SMILES strings for existing drugs as input, with the matching indications of the drug as the target output. The indication-to-drug task takes the set of indications for a drug as input and seeks to generate the corresponding SMILES string for a drug that treats the listed conditions.

We employ all available MolT5 model sizes for our experiments and evaluate them separately across the two datasets. Additionally, we perform the experiments under three different configurations:

1. Evaluation of the baseline models on the entire available dataset
2. Evaluation of the baseline models on 20% of the dataset
3. Fine-tuning the models on 80% of the dataset followed by evaluation on the 20% subset

We found that larger MolT5 models outperformed the smaller ones across all configurations and tasks. It should also be noted that fine-tuning MolT5 models has a negative impact on the performance.

Following these preliminary experiments, we train the smallest available MolT5 model from scratch using a custom tokenizer. This custom model performed better on DrugBank data than on ChEMBL data on the drug-to-indication task, perhaps due to a stronger signal between the drug indications and SMILES strings in their dataset, owing to the level of detail in their indication descriptions. Fine-tuning the custom model on 80% of either dataset did not degrade model performance for either task, and some metrics saw improvement due to fine-tuning. Overall, fine-tuning for the indication-to-drug task did not consistently improve the performance, which holds for both ChEMBL and DrugBank datasets.

While the performance of the custom tokenizer approach is still not satisfying, there is promise in using a larger model and having access to more data. If we have a wealth of high-quality data to train models on translation between drugs and their indications, it may become possible to improve performance and facilitate novel drug discovery with LLMs.

In this paper, we make the following contributions:

1. We introduce a new task: translation between drug molecules and indications.
2. We conduct various experiments with T5-based LLMs and two datasets (DrugBank and ChEMBL). Our experiments consider 16 evaluation metrics across all experiments. In addition, we discuss the current bottlenecks that, if addressed, have the potential to significantly improve the performance on the task.

## Results

### Evaluation of MolT5 models

We performed initial experiments using MolT5 models from HuggingFace (GitHub links: https://huggingface.co/laituan245/molt5-small/tree/main, https://huggingface.co/laituan245/molt5-base/tree/main, https://huggingface.co/laituan245/molt5-large/tree/main). MolT5 offers three model sizes and fine-tuned models of each size, which support each task of our experiments. For experiments generating SMILES strings from drug indications (drug-to-indication), we used the fine-tuned models MolT5-smiles-to-caption, and for generating SMILES strings from drug indications (indication-to-drug), we used the models MolT5-caption-to-smiles. For each of our Tables, we use the following flags: FT (denotes experiments where we fine-tuned the models on 80% of the dataset and evaluated on the remaining 20% test subset), SUB (denotes experiments where the models are evaluated solely on the 20% test subset), and FULL (for experiments evaluating the models on the entirety of each dataset).

**Table 1:** Evaluation metrics used in the experiments.

Metric	Description	Values	Direction
BLEU^46^	Computes similarity as geometric mean of n-gram precisions scaled by brevity penalty	$[0, 1]$	$\uparrow$
Exact	Represents whether the string match is exact (1) or not (0)	{0, 1}	$\uparrow$
Levenshtein^47^	Measures *Levenshtein* edit distance between two strings	$[0, \infty )$	$\downarrow$
MACCS^48,49^	Computes Tanimoto similarity between two molecular *MACCS* fingerprints	$[0, 1]$	$\uparrow$
RDK^48,50^	Computes Tanimoto similarity between two molecular *RDK* fingerprints	$[0, 1]$	$\uparrow$
Morgan^48,51^	Computes Tanimoto similarity between two molecular *Morgan* fingerprints	$[0, 1]$	$\uparrow$
FCD^52^	Measures distance between distributions of real-world and LLM-generated molecules	$[0, \infty )$	$\uparrow$
Text2Mol^53^	Uses pretrained model to compute similarity between SMILES string and text	$[0, 1]$	$\uparrow$
Validity	Represents whether the generated SMILES string is syntactically valid (1) or not (0)	$\{0, 1\}$	$\uparrow$
BLEU-2^46^	Computes cumulative 2-gram *BLEU* score	$[0, 1]$	$\uparrow$
BLEU-4^46^	Computes cumulative 4-gram *BLEU* score	$[0, 1]$	$\uparrow$
ROUGE-1^54,55^	Measures overlap of unigrams between the candidate and reference strings	$[0, 1]$	$\uparrow$
ROUGE-2^54,55^	Measures overlap of bigrams between the candidate and reference strings	$[0, 1]$	$\uparrow$
ROUGE-L^54,56^	Calculates similarity via Longest Common Subsequence (LCS) statistics	$[0, 1]$	$\uparrow$
METEOR^57^	Computes similarity between two strings via weighted unigram F-score	$[0, 1]$	$\uparrow$
Text2Mol^53^	Uses a pretrained model to compute similarity between two strings	$[0, 1]$	$\uparrow$

$\uparrow$: higher values result in higher string similarity.$\downarrow$: higher values result in lower string similarity.

For evaluating drug-to-indication, we employ the natural language generation metrics BLEU^46^, ROUGE^54–56^, and METEOR^57^, as well as the Text2Mol^53^ metric, which generates similarities of SMILES-Indication pairs. As for evaluation of indication-to-drug, we measure exact SMILES string matches, Levenshtein distance^47^, SMILES BLEU scores, the Text2Mol similarity metric, as well as three different molecular fingerprint metrics: MACCS^48,49^, RDK^48,50^, and Morgan FTS^48,51^, where FTS stands for fingerprint Tanimoto similarity^48^, as well as the proportion of returned SMILES strings that are valid molecules. The final metric for evaluating SMILES generation is FCD, or Fréchet ChemNet Distance, which measures the distance between two distributions of molecules from their SMILES strings^52^. Table 1 presents both drug-to-indication and indication-to-drug metrics, including their descriptions, values, and supported intervals.

Table 2 lists four examples of inputs and our model outputs for both drug-to-indication and indication-to-drug tasks using the large MolT5 model and ChEMBL data. Molecular validity is determined through the use of RDKit (https://www.rdkit.org/docs/index.html), an open-source toolkit for cheminformatics, with the reason for invalidity given. Indication quality is determined by the Text2Mol string similarity between the ground truth and generated indications. We can observe that the proposed model could output valid molecules using SMILES strings for a given indication, and output meaningful indication, such as cancer, for a given molecule. However, there are some misspelling issues in the generated indication due to the small size of T5 model. We hypothesize that LLMs with larger size of parameters could significantly improve the validity of the generated molecules and indications.

**Table 2:** First four rows: example SMILES strings from the indication-to-drug task; Last four rows: example MolT5 indication generations from the drug-to-indication task.

Input	Ground truth	Output	Validity/similarity
Indication-to-drug			
Diabetes mellitus	COCCOc1cnc(NS(=O)(=O)c2ccccc2)nc1	O=C([O-])CC(=O)[O-]	Valid
Coronary artery disease	CCOC(=O)C(C)=O	CCCCC[C@H](O)CC=CCC=CCCCC(=O)O	Valid
Respiratory system disease	CCC1(C)CC(=O)NC(=O)C1	[H+].C(=O)[O-])[O-]	Syntax Error
Hemorrhage	CC1=CC(=O)c2ccccc2C1=O	C(=O)C(=O)O)O.O)O.O)O.O.O	Syntax Error
Drug-to-indication			
CN(C)CCOC(c1ccccc1)c1ccccc1	Allergic disease ... cancer ... eczema ...	... and cancer ...	0.2206
CCc1cc(C(N)=S)ccn1	Multidrug-resistant tuberculosis osteomyelitis ...	... cancer	0.2262
O=C([O-])c1ccccc1.[Na+]	Encephalopathy psychosis	Inamideamide protein protein proteinamide.	0.0183
Clc1ccccc1CN1CCc2sccc2C1	Internal carotid artery stenosis ... Recurrent thrombophlebitis	Amideamideamide.	0.0316

**Table 3:** DrugBank drug-to-indication results.

Model	BLEU-2	BLEU-4	ROUGE-1	ROUGE-2	ROUGE-L	METEOR	Text2Mol
FT-small	0.0013	0.0000	0.0011	0.0000	0.0011	0.011	0.0805
SUB-small	**0.0224**	**0.0053**	**0.0982**	**0.0068**	**0.0809**	**0.1007**	0.2368
FULL-small	0.0213	0.0036	0.0965	0.0061	0.0801	0.0987	**0.3234**
FT-base	0.0006	0.0000	0.0004	0.0000	0.0004	0.0092	0.0683
SUB-base	**0.0227**	**0.0053**	**0.0973**	**0.0073**	**0.0808**	**0.1020**	**0.3317**
FULL-base	0.0208	0.0034	0.0966	0.0059	0.0803	0.0992	0.3217
FT-large	0.0006	0.0000	0.0007	0.0000	0.0007	0.0110	0.0532
SUB-large	**0.0298**	**0.0097**	**0.1015**	**0.0115**	**0.0835**	**0.1167**	**0.5001**
FULL-large	0.0281	0.0080	0.1007	0.0098	0.0814	0.1127	0.4864

Significant values are in [boldunderlined].

**Table 4:** ChEMBL drug-to-indication results.

Model	BLEU-2	BLEU-4	ROUGE-1	ROUGE-2	ROUGE-L	METEOR	Text2Mol
FT-small	0.0000	0.0000	0.0011	0.0000	0.0011	0.0017	0.1070
SUB-small	0.0005	0.0000	0.0032	0.0000	0.0029	**0.0079**	**0.3353**
FULL-small	**0.0005**	0.0000	**0.0033**	0.0000	**0.0032**	0.0078	0.3237
FT-base	0.0000	0.0000	0.0012	0.0000	0.0012	0.0026	0.0799
SUB-base	0.0005	0.0000	0.0033	0.0000	0.0032	0.0076	**0.3315**
FULL-base	**0.0007**	0.0000	**0.0034**	0.0000	**0.0033**	**0.0078**	0.3171
FT-large	0.0000	0.0000	0.0011	0.0000	0.0011	0.0010	0.0917
SUB-large	**0.0021**	**0.0007**	0.0052	**0.0007**	0.0049	**0.0118**	**0.4903**
FULL-large	0.0019	0.0006	**0.0053**	**0.0007**	**0.0050**	**0.0118**	0.4830

Significant values are in [boldunderlined].

Tables 3 and 4 show the results of MolT5 drug-to-indication experiments on DrugBank and ChEMBL data, respectively. Larger models tended to perform better across all metrics for each experiment. Across almost all metrics for the drug-to-indication task, on both DrugBank and ChEMBL datasets, the model performed the best on the 20% subset data. At the same time, both the subset and full dataset evaluations yielded better results than fine-tuning experiments. As MolT5 models are trained on molecular captions, fine-tuning using indications could introduce noise and weaken the signal between input and target text. The models performed better on DrugBank data than ChEMBL data, which may be due to the level of detail provided by DrugBank for their drug indications.

**Table 5:** DrugBank indication-to-drug results.

Model	BLEU	Exact	Levenshtein	MACCS	RDK	Morgan	FCD	Text2Mol	Validity
FT-small	0.0020	0.0000	**77.0375**	0.0408	0.0023	0.0241	0.0000	0.0000	0.0017
SUB-small	0.1524	0.0000	89.3278	0.2747	**0.1729**	**0.1026**	0.0000	**0.1663**	**0.3661**
FULL-small	**0.1627**	**0.0003**	87.0366	**0.2822**	0.1644	0.0992	**11.2862**	0.0645	0.3628
FT-base	0.0002	0.0000	640.9418	0.0000	0.0000	0.0000	**0.0000**	0.0000	0.0017
SUB-base	0.1563	0.0000	**92.9151**	**0.3147**	0.1898	**0.1214**	0.0000	0.1220	**0.3278**
FULL-base	**0.1614**	**0.0003**	95.0343	0.3145	**0.1933**	0.1177	**11.2079**	**0.1472**	0.3106
FT-large	0.0000	0.0000	1315.0585	0.0000	0.0000	0.0000	**0.0000**	0.1472	0.0000
SUB-large	0.1314	**0.0166**	**113.3877**	0.3907	0.2758	0.1673	0.0000	**0.2972**	**0.5655**
FULL-large	**0.1375**	0.0163	114.6298	**0.3982**	**0.2819**	**0.1709**	**5.5990**	0.2516	0.5462

Significant values are in [boldunderlined].

**Table 6:** ChEMBL indication-to-drug results.

Model	BLEU	Exact	Levenshtein	MACCS	RDK	Morgan	FCD	Text2Mol	Validity
FT-small	0.0401	0.0000	**84.3199**	0.0571	0.0094	0.0070	0.0000	0.0000	0.0142
SUB-small	**0.1190**	0.0000	126.1835	0.2387	0.1162	0.0629	0.0000	**0.0395**	**0.3246**
FULL-small	0.1114	0.0000	132.5282	**0.2442**	**0.1247**	**0.0656**	**19.6213**	0.0219	0.3199
FT-base	0.0203	0.0000	516.0212	0.1235	0.0237	0.0325	0.0000	0.0000	0.0098
SUB-base	**0.1956**	0.0000	**76.7455**	**0.2997**	0.1878	**0.0945**	0.0000	0.0566	**0.3662**
FULL-base	0.1935	0.0000	77.3259	0.2996	**0.1924**	0.0922	**19.6774**	**0.0620**	0.3404
FT-large	0.0115	0.0000	339.3972	0.0000	0.0000	0.0000	0.0000	0.0620	0.0000
SUB-large	**0.0699**	0.0000	**276.5310**	**0.3590**	0.2613	**0.0851**	0.0000	**0.1934**	0.3140
FULL-large	0.0684	0.0000	280.9910	0.3559	**0.2626**	0.0830	**16.3108**	0.0482	**0.3199**

Significant values are in [boldunderlined].

Tables 5 and 6 show the results of MolT5 indication-to-drug experiments on DrugBank and ChEMBL data, respectively. The tables indicate that fine-tuning the models on the new data worsens performance, reflected in FT experiments yielding worse results than SUB or FULL experiments. Also, larger models tend to perform better across all metrics for each experiment.

In our drug-to-indication and indication-to-drug experiments, we see that fine-tuning the models causes the models to perform worse across all metrics. Additionally, larger models perform better on our tasks. However, in our custom tokenizer experiments, we pretrain MolT5-Small without the added layers of SMILES-to-caption and caption-to-SMILES. By fine-tuning the custom pretrained model on our data for drug-to-indication and indication-to-drug tasks, we aim to see improved results.

### Evaluation of custom tokenizer

**Table 7:** Results for MolT5 augmented with custom tokenizer, drug-to-indication.

Model	BLEU-2	BLEU-4	ROUGE-1	ROUGE-2	ROUGE-L	METEOR	Text2Mol
FT-DrugBank	**0.0006**	0.0000	**0.0013**	0.0000	**0.0013**	**0.0141**	**0.0706**
FT-ChEMBL	0.0000	0.0000	0.0011	0.0000	0.0011	0.0017	0.0699
SUB-DrugBank	**0.0008**	0.0000	**0.0014**	0.0000	**0.0013**	**0.0137**	0.0811
SUB-ChEMBL	0.0000	0.0000	0.0012	0.0000	0.0012	0.0012	**0.0836**
FULL-DrugBank	**0.0010**	0.0000	0.0014	0.0000	0.0014	**0.0133**	0.0787
FULL-ChEMBL	0.0000	0.0000	**0.0016**	0.0000	**0.0016**	0.0014	**0.0868**

Significant values are in [boldunderlined].

**Table 8:** Results for MolT5 augmented with custom tokenizer, indication-to-drug.

Model	BLEU	Exact	Levenshtein	MACCS	RDK	Morgan	FCD	Text2Mol	Validity
FT-DrugBank	**0.0154**	0.0000	**174.0865**	0.0440	**0.0354**	**0.0513**	0.0000	0.0000	0.0050
FT-ChEMBL	0.0136	0.0000	454.1142	**0.1455**	0.0233	0.0327	0.0000	0.0000	**0.0073**
SUB-DrugBank	0.0175	0.0000	**170.0050**	0.0452	0.0140	**0.0532**	0.0000	0.0000	0.0067
SUB-ChEMBL	**0.0252**	0.0000	281.2072	**0.0605**	**0.0239**	0.0493	0.0000	**0.1989**	**0.0090**
FULL-DrugBank	0.0174	0.0000	**175.9574**	0.0825	**0.0552**	**0.0532**	0.0000	0.0728	**0.0087**
FULL-ChEMBL	**0.0234**	0.0000	286.5869	**0.1180**	0.0449	0.0356	0.0000	**0.2707**	0.0072

Significant values are in [boldunderlined].

Tables 7 and 8 show the evaluation of MolT5 pretrained with the custom tokenizer on the drug-to-indication and indication-to-drug tasks, respectively. For drug-to-indication, the model performed better on the DrugBank dataset, reflected across all metrics. This performance difference may be due to a stronger signal between drug indication and SMILES strings in the DrugBank dataset, as the drug indication contains more details. Fine-tuning the model on 80% of either of the datasets did not worsen the performance for drug-to-indication as it did in the baseline results, and some metrics showed improved results. The results for indication-to-drug are more mixed. The model does not consistently perform better across either dataset and fine-tuning the model affects the evaluation metrics inconsistently.

## Discussion

In this paper, we proposed a novel task of translating between drugs and indications, considering both drug-to-indication and indication-to-drug subtasks. We focus on generating indications from the SMILES strings of existing drugs and generating SMILES strings from sets of indications. Our experiments are the first attempt at tackling this problem. After conducting experiments with various model configurations and two datasets, we hypothesized potential issues that need further work. We believe that properly addressing these issues could significantly improve the performance of the proposed tasks.

The signal between SMILES strings and indications is poor. In the original MolT5 task (translation between molecules and their textual descriptions), “similar” SMILES strings often had similar textual descriptions. In the case of drug-to-indication and indication-to-drug tasks, similar SMILES strings might have completely different textual descriptions as they are different drugs, and their indications also differ. One could also make a similar observation about SMILES strings that are different: drug indications may be similar. Having no direct relationships between drugs and indications makes it hard to achieve high performance on proposed tasks. We hypothesize that having an intermediate representation that drugs (or indications) map to may improve the performance. As an example, mapping a SMILES string to its caption (MolT5 task) and then caption to indication may be a potential future direction of research.

The signal between drugs and indications is not the only issue: the data is also scarce. Since we do not consider random molecules and their textual descriptions but drugs and their indications, the available data is limited by the number of drugs. In the case of both ChEMBL and DrugBank datasets, the number of drug-indication pairs was under 10000, with the combined size also being under 10000. Finding ways to enrich data may help establish a signal between SMILES strings and indications and could be a potential future avenue for exploration.

Overall, the takeaway from our experiments is that larger models tend to perform better. By using a larger model and having more data (or data that has a stronger signal between drug indications and SMILES strings), we may be able to successfully translate between drug indications and molecules (i.e., SMILES strings) and ultimately facilitate novel drug discovery.

We note that our experiments did not involve human evaluation of the generated indications and relied entirely on automated metrics. We acknowledge that such metrics may not correlate well with human judgment^58–60^. At the same time, manually reviewing thousands of indications would have been expensive and would involve a lot of human labor. Future work could potentially consider incorporating humans in the loop or using LLMs to assess the quality of generated indications.

Experiments with other models and model architectures can be another avenue for exploration. Some potential benefits may include better performance, lower latency, and improved computational complexity. As an example, our current method uses the transformer architecture, which has the overall time complexity of $\text {O}(n^2 \cdot d + n \cdot d^2)$ (where $n$ is the sequence length and $d$ is the embedding dimension), with $\text {O}(n^2 \cdot d)$ being the time complexity of the attention layer alone. On the other hand, State Space Models (SSMs), such as Mamba^61^, scale linearly with the sequence length.

## Methods

**Figure 1:**
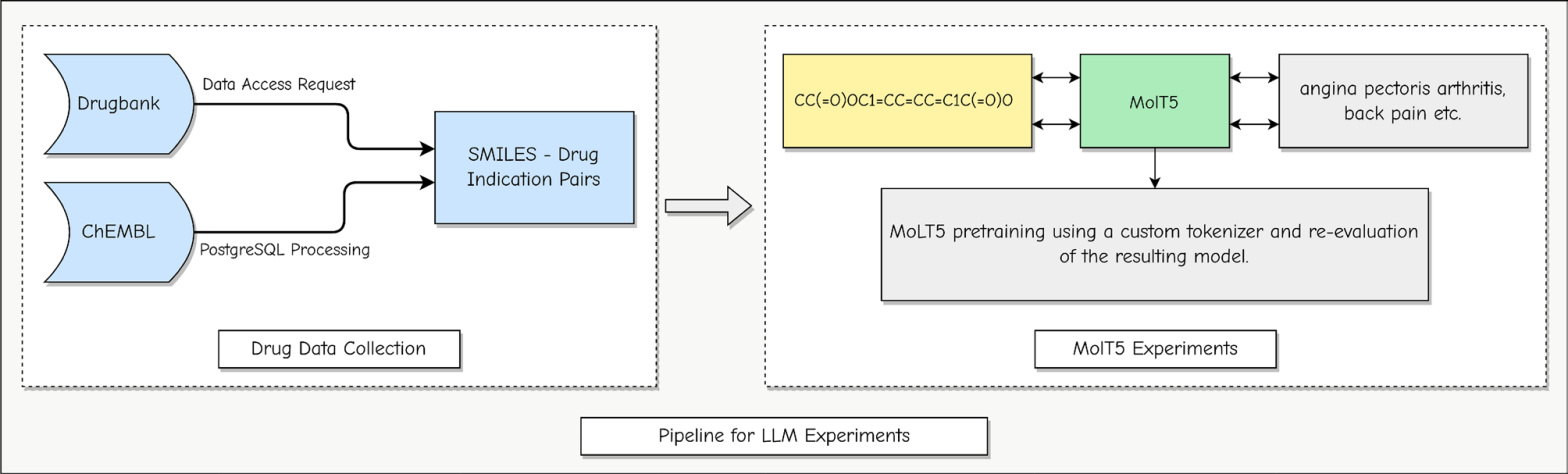
Overview of the methodology of the experiments: drug data is compiled from ChEMBL and DrugBank and utilized as input for MolT5. Our experiments involved two tasks: drug-to-indication and indication-to-drug. For the drug-to-indication task, SMILES strings of existing drugs were used as input, producing drug indications as output. Conversely, for the indication-to-drug task, drug indications of the same set of drugs were the input, resulting in SMILES strings as output. Additionally, we augmented MolT5 with a custom tokenizer in pretraining and evaluated the resulting model on the same tasks.

This section describes the dataset, analysis methods, ML models, and feature extraction techniques used in this study. Figure 1 shows the flowchart of the process. We adjust the workflow of existing models for generating molecular captions to instead generate indications for drugs. By training LLMs on the translation between SMILES strings and drug indications, we endeavor to one day be able to create novel drugs that treat medical conditions.

### Data

Our data comes from two databases, DrugBank^62^ and ChEMBL^63^, which we selected due to the different ways they represent drug indications. DrugBank gives in-depth descriptions of how each drug treats patients, while ChEMBL provides a list of medical conditions each drug treats. Table 9 outlines the size of each dataset, as well as the length of the SMILES and indication data. In the case of DrugBank, we had to request access to use the drug indication and SMILES data. The ChEMBL data was available without request but required establishing a database locally to query and parse the drug indication and SMILES strings into a workable format. Finally, we prepared a pickle file for both databases to allow for metric calculation following the steps presented in MolT5^26^.

**Table 9:** Dataset Details.

Dataset statistic	DrugBank	ChEMBL
Number of drug-Indication pairs	3004	6127
Minimum indication length (characters)	19	34
Minimum SMILES length (characters)	1	1
Average indication length (characters)	259	114
Average SMILES length (characters)	59	67
Maximum indication length (characters)	3517	524
Maximum SMILES length (characters)	710	1486

### Models

We conducted initial experiments using the MolT5 model, based on the T5 architecture^26^. The T5 basis of the model gives it textual modality from pretraining on the natural language text dataset Colossal Clean Crawled Corpus (C4)^64^, and the pretraining on 100 million SMILES strings from the ZINC-15 dataset^65^ gives the model molecular modality.

In our experiments, we utilized fine-tuned versions of the available MolT5 models: SMILES-to-caption, fine-tuned for generating molecular captions from SMILES strings, and caption-to-SMILES, fine-tuned for generating SMILES strings from molecular captions. However, we seek to evaluate the model’s capacity to translate between drug indications and SMILES strings. Thus, we use drug indications in the place of molecular captions, yielding our two tasks: drug-to-indication and indication-to-drug.

The process of our experiments begins with evaluating the baseline MolT5 model for each task on the entirety of the available data (3004 pairs for DrugBank, 6127 pairs for ChEMBL), on a 20% subset of the data (601 pairs for DrugBank, 1225 pairs for ChEMBL), and then fine-tuning the model on 80% (2403 pairs for DrugBank, 4902 pairs for ChEMBL) of the data and evaluating on that same 20% subset.

After compiling the results of the preliminary experiments, we decided to use a custom tokenizer with the MolT5 model architecture. While the default tokenizer leverages the T5 pretraining, the reason is that treating SMILES strings as a form of natural language and tokenizing it accordingly into its component bonds and molecules could improve model understanding of SMILES strings and thus improve performance.

### MolT5 with custom tokenizer

**Figure 2:**
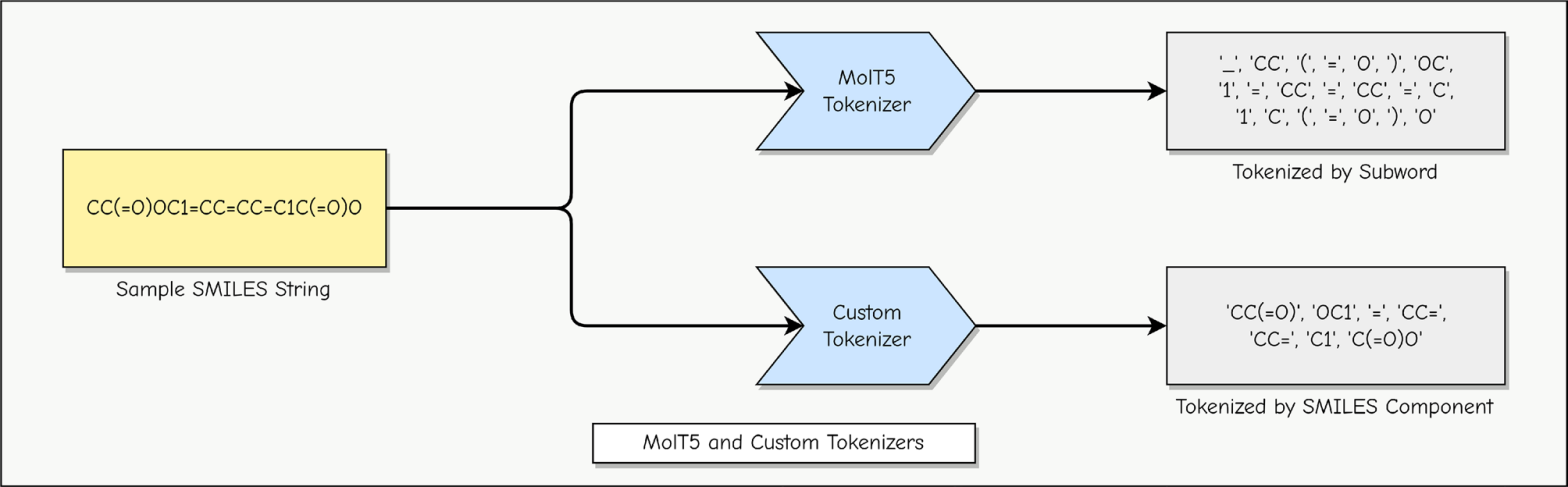
MolT5 and custom tokenizers: MolT5 tokenizer uses the default English language tokenization and splits the input text into subwords. The intuition is that SMILES strings are composed of characters typically found in English text, and pretraining on large-scale English corpora may be helpful. On the other hand, the custom tokenizer method utilizes the grammar of SMILES and decomposes the input into grammatically valid components.

The tokenizer for custom pretraining of MolT5 that we selected came from previous work on adapting transformers for SMILES strings^66^. This tokenizer separates SMILES strings into individual bonds and molecules. Figure 2 illustrates the behavior of both MolT5 and custom tokenizers. Due to computational limits, we only performed custom pretraining of the smallest available MolT5 model, with 77 million parameters. Our pretraining approach utilized the model configuration of MolT5 and JAX (https://jax.readthedocs.io/en/latest/index.html) / Flax (https://github.com/google/flax) to execute the span-masked language model objective on the ZINC dataset^64^. Following pretraining, we assessed model performance on both datasets. The experiments comprised three conditions: fine-tuning on 80% (2403 pairs for DrugBank, 4902 pairs for ChEMBL) of the data and evaluating on the remaining 20% (601 pairs for DrugBank, 1225 pairs for ChEMBL), evaluating on 20% of the data without fine-tuning, and evaluating on 100% (3004 pairs for DrugBank, 6127 pairs for ChEMBL) of the data.

## Data Availability

ChEMBL and DrugBank datasets are publicly available.
